# The validity and reliability of the “*My Jump App*” for measuring jump height of the elderly

**DOI:** 10.7717/peerj.5804

**Published:** 2018-10-15

**Authors:** Rejane Maria Cruvinel-Cabral, Iransé Oliveira-Silva, André Ricarte Medeiros, João Gustavo Claudino, Pedro Jiménez-Reyes, Daniel A. Boullosa

**Affiliations:** 1 Physical Education, Catholic University of Brasilia, Águas Claras, Brazil; 2 Physical Education Course, University Center of Anápolis - UniEVANGÉLICA, Anápolis, Brazil; 3 School of Physical Education and Sport, Laboratory of Biomechanics, University of São Paulo, São Paulo, Brazil; 4 Centre for Sport Studies, King Juan Carlos University, Móstoles, Spain

**Keywords:** Explosiveness, Functional capacity, Physical capacity, Older, Seniors

## Abstract

**Background:**

The ability to jump has been related to muscle strength and power, speed and amplitude of the lower limbs movements, and specifically for the elderly, the vertical jump has been shown to be a good predictor of functional capacity and risk of falling. The use of a mobile application (*App*) which can measure the vertical jump (i.e., iPhone *App My Jump*) has recently emerged as a simple, cheap and very practical tool for evaluation of jump ability. However, the validity of this tool for the elderly population has not been tested yet. The elderly usually perform very low jumps and therefore the signal-to-noise ratio may compromise the validity and reliability of this method. Thus, the aim of the current study was to verify the validity and reliability of the iPhone *App* “*My Jump*” for the evaluation of countermovement jump (CMJ) height within an elderly population.

**Methods:**

After familiarization, 41 participants performed three CMJs assessed via a contact mat and the *My Jump App*. The intraclass correlation coefficient (ICC) was used to verify the relative reliability, while the coefficient of variation (CV%) and the typical error of measurement (TEM) were used to verify the absolute reliability. Pearson’s correlation coefficient was used to verify the strength of the relationship between methods (i.e., concurrent validity), a Bland–Altman plot to show their agreement, and the Student’s *t*-test to identify systematic bias between them. For reliability analyses, all jumps were considered (i.e., 123). All jumps (i.e., 123), the average height of each attempt (i.e., 41), and the highest jump, were considered for validity analyses.

**Results:**

The CMJ height of the highest jump was 10.78 ± 5.23 cm with contact mat, and 10.87 ± 5.32 with *My Jump App*, with an identified systematic bias of 0.096 cm (*P* = 0.007). There was a nearly perfect correlation between methods (*r* = 0.999; *P* = 0.000, in all cases) with a very good agreement observed (0.3255 to −0.5177 cm, 0.2797 to −0.5594 cm, and 0.3466 to −0.6264 cm, for highest jump height, average jump height, and all jump heights, respectively). The ICC of the *My Jump App* was 0.948, the TEM was 1.150 cm, and the CV was 10.10%.

**Conclusion:**

Our results suggest that the *My Jump App* is a valid and reliable tool compared to the contact mat for evaluating vertical jump performance in the elderly. Therefore, it allows a simple and practical assessment of lower limbs’ power in this population. For the elderly, as well as for other populations with low jumping heights, the highest jump height and the average jump height could be used indistinctly.

## Introduction

The vertical jump is a capacity of great interest and has been widely used as a measure of neuromuscular performance in professional (Klavora, 2000; Watkins et al., 2017) and recreational athletes (Driller et al., 2017; Boullosa et al., 2018), and in the elderly population among others (Petersen et al., 2007; Pijnappels et al., 2008). It is an indirect indicator of explosive muscular strength of the lower limbs (Balsalobre-Fernández, Glaister & Lockey, 2015; Markovic, 2007). The ability to jump has also been related to muscle strength and power, as well as speed and amplitude of lower limbs movements (Pijnappels et al., 2008).

Specifically, for the elderly, the vertical jump has been shown to be a good predictor of functional capacity (Pijnappels et al., 2008), and risk of falling (Lee, Biggan & Ray, 2016). It has been previously shown that loss of functional capacity interferes with the performance of activities of daily living (ADL) (Foldvari et al., 2000; Macaluso & De Vito, 2004; Farias et al., 2013), decreasing the independence in ADL, and also increasing the prevalence of falls (Lee, Biggan & Ray, 2016; Vieira et al., 2013). Therefore, it is reasonable to highlight that the vertical jump can be one of the most appropriate, useful, and suitable tests to evaluate the functional capacity of elderly people.

The instruments traditionally used for measuring the vertical jumps have been force platforms, infrared cells, video recording techniques, linear position transducers, and contact mats, which present good validity and accuracy/reliability in a variety of jumping techniques (Bosquet, Berryman & Dupuy, 2009; Carlos-Vivas et al., 2016; Pueo et al., 2017; Whitmer et al., 2015; Haynes et al., 2018). However, these instruments are relatively expensive and not easy to transport (Balsalobre-Fernández, Glaister & Lockey, 2015; Gallardo-Fuentes et al., 2016). This reality has recently changed with the use of a mobile application (*App*), which can measure the vertical jump height (i.e., iPhone *App My Jump*) (Balsalobre-Fernández, Glaister & Lockey, 2015; Carlos-Vivas et al., 2016; Driller et al., 2017). This *App* is very cheap and very practical to use in the most diverse situations (Driller et al., 2017; Haynes et al., 2018). The validity of this *App* has been tested for the young (Balsalobre-Fernández, Glaister & Lockey, 2015; Driller et al., 2017; Gallardo-Fuentes et al., 2016), presenting very good accuracy for jumping performance higher than ∼25 cm. However, the validity and reliability of this tool for the elderly population have not been tested yet. This is important since the elderly usually perform jumps lower than ∼20 cm (Farias et al., 2013) and therefore the signal-to-noise ratio may compromise the validity and reliability of this method in the elderly as well as other populations with low jumping values. Thus, the aim of this study was to verify the validity and reliability of the iPhone *App My Jump* for the evaluation of countermovement jump (CMJ) height of the elderly when compared to a contact mat, a previously validated method for other population (Balsalobre-Fernández, Glaister & Lockey, 2015; Yingling et al., 2018).

## Materials and Methods

### Study design

A sample of elderly people of both sexes was tested, after familiarization, with three maximal CMJs in the same day. All jumps (i.e., 123 jumps) measured with the *App* and the reference method (i.e., contact mat) as well as the highest jump and the average of the three attempts were used for concurrent validity analysis. The three jump heights measured with the *App* were used for intraday reliability analyses.

### Participants

A total of 41 participants, 12 men (73.2 ± 6.4 years; 68.3 ± 12.7 kg) and 29 women (69.4 ± 8.9 years; 64.7 ± 12.6 kg), volunteered to participate in this study. A total of 13 were living in institutions while 28 were living with their families. Individuals who (a) were diagnosed with chronic obstructive pulmonary disease, (b) presented any form of intense pain, (c) or did not exhibit walking independence, were excluded from participation. After receiving information of the procedures, all of them signed an informed written consent. The study protocol adhered to the Code of Ethics of the World Medical Association (Declaration of Helsinki) and was approved by the Institutional Review Board (1.001.775).

### Procedures

All participants were familiarized with CMJ technique before testing. The CMJ technique involved the participants standing in a fully extended position and feet approximately shoulder-width apart. Subsequently, they were instructed to jump as high as possible after performing a countermovement with the same take-off and landing positions. All participants performed four jumps in this familiarization session.

Jump testing was performed on a separate day between 8 and 10 a.m. after measurement of the volunteer body mass and warming up. Body mass was measured in a fasted state to the nearest 0.1 kg with a calibrated scale (PL 200; Filizola, São Paulo, Brazil). A standard 5 min warm-up composed of some calisthenics (sit-ups and sit-downs, ballistic movements in different positions) and three submaximal CMJs was subsequently performed. Then, each participant performed three maximal CMJs on a custom-made contact mat connected to a computer with specific software (Chronojump, version. 1.6.2; Boscosystem, Barcelona, Spain). This equipment has been previously demonstrated to be valid and reliable (Pagaduan & De Blas, 2004; De Blas et al., 2012). At the same time, the jump was recorded by the same researcher with a mobile (cell) phone (iPhone 7; Apple, Cupertino, CA, USA) at a sampling rate of 240 Hz, using the *My Jump App*.

### Statistical analysis

Descriptive data are presented as mean ± SD. Normality was tested using the Shapiro–Wilk test. The homoscedasticity was verified with a scatter plot and with the Levene’s test when necessary. There is an inability to calculate reliability values when using the highest jump, due to the need for at least two measurements for the calculations. Therefore, the intraclass correlation coefficient (ICC; 2, 1) was applied to the three jumps to measure the relative reliability of the *My Jump App*. The absolute reliability of the *App* was calculated using the typical error of measurement (TEM) as the square root of the mean square error term from the ANOVA reported in absolute units, as well as coefficient of variation (CV%) (Weir, 2005). For concurrent validity, the analysis were performed using the highest jump (i.e., 41 jumps), the average of the three attempts (i.e., 41 jumps), and all jumps (i.e., 123) for a higher statistical power. For this purpose, Pearson’s correlation coefficient was used to verify the strength of the relationship between methods. Subsequently, a Bland–Altman plot was used to show their agreement (Bland & Altman, 1986), and a student’s *t*-test was performed to verify if there was a significant bias between methods. The statistical significance level was set at *P* < 0.05. Statistical analyses were performed with a statistical package (IBM SPSS version 22.0; SPSS, Chicago, IL, USA).

## Results

The vertical jump heights in all attempts, the highest jump height, and the average of the three attempts measured with the two methods are presented in Table 1.

**Table 1:** Mean ± SD for jump heights recorded with the two methods.

	Contact mat	*My Jump App*	Student’s *P*	Pearson’s r (*P*)
All jumps height (cm)	10.01 ± 5.08	10.15 ± 5.16	0.000	0.999 (0.000)
Average jump height (cm)	10.01 ± 5.03	10.15 ± 5.11	0.000	0.999 (0.000)
Highest jump height (cm)	10.78 ± 5.23	10.87 ± 5.32	0.007	0.999 (0.000)
First jump height (cm)	9.78 ± 4.78	9.90 ± 4.86	0.001	0.999 (0.000)
Second jump height (cm)	9.88 ± 5.28	9.99 ± 5.33	0.001	0.999 (0.000)
Third jump height (cm)	10.36 ± 5.27	10.55 ± 5.39	0.000	0.999 (0.000)

**Figure 1:**
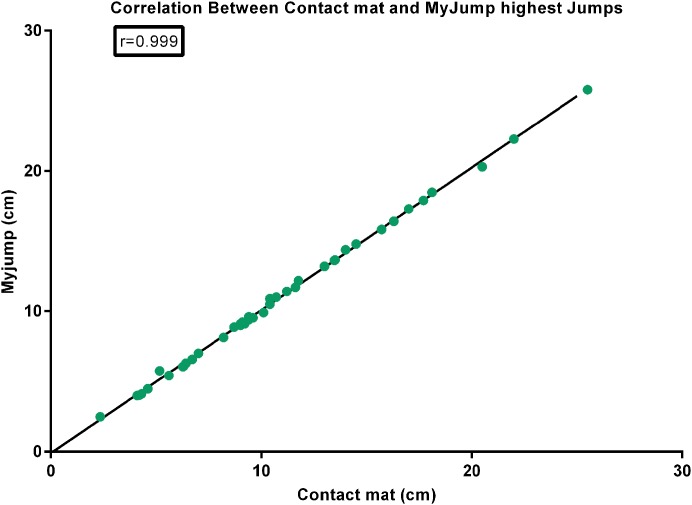
Correlation between contact mat and *My Jump* highest jumps.

**Figure 2:**
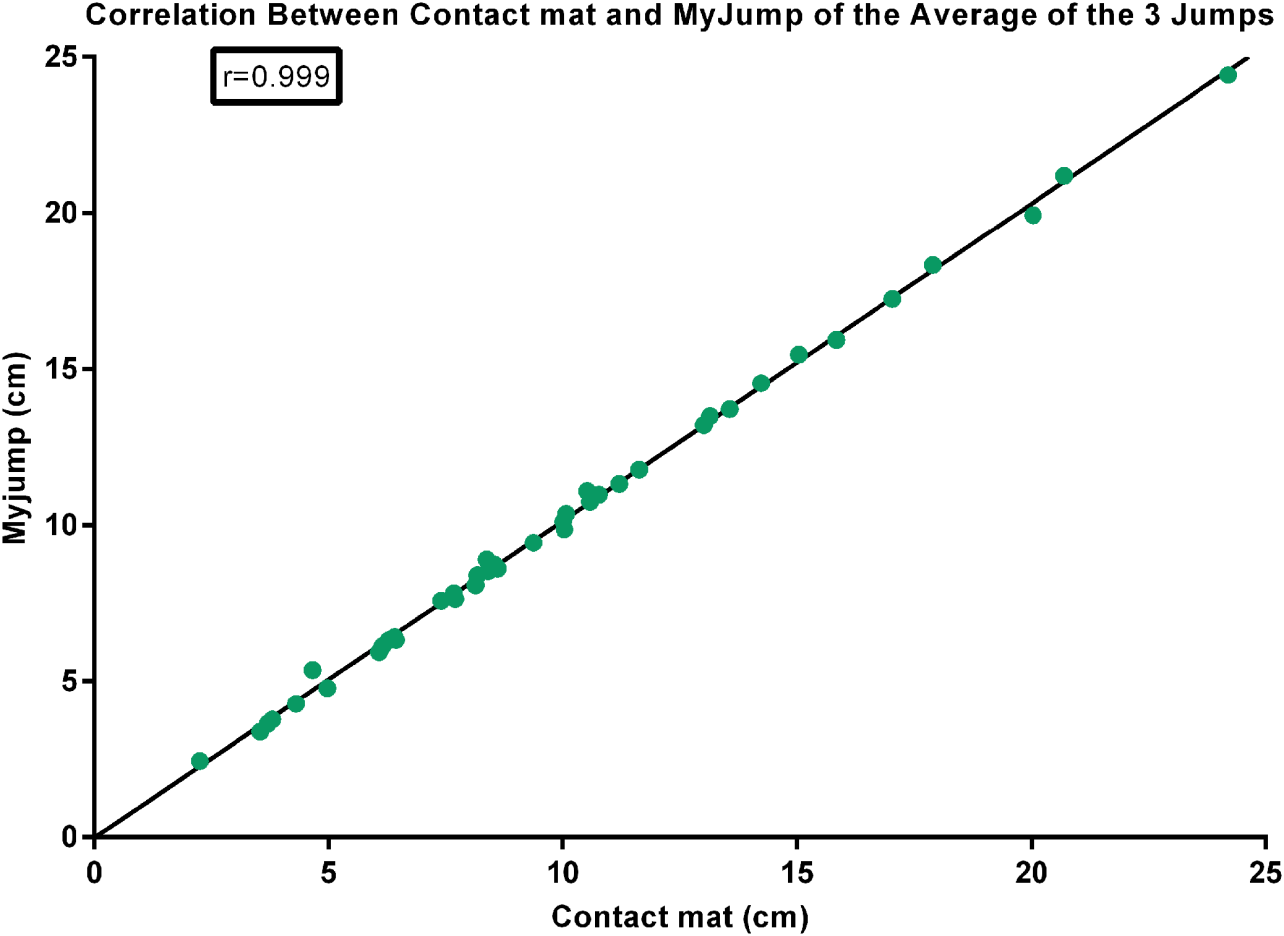
Correlation between contact mat and *My Jump* of the average of the three jumps.

**Figure 3:**
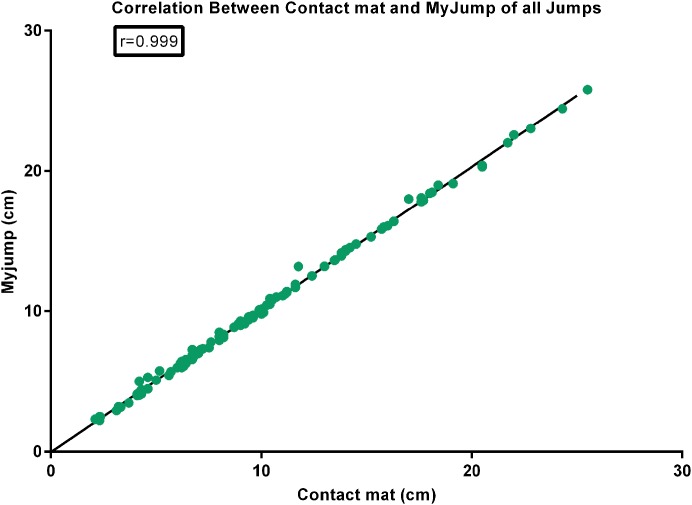
Correlation between contact mat and *My Jump* of all jumps.

**Figure 4:**
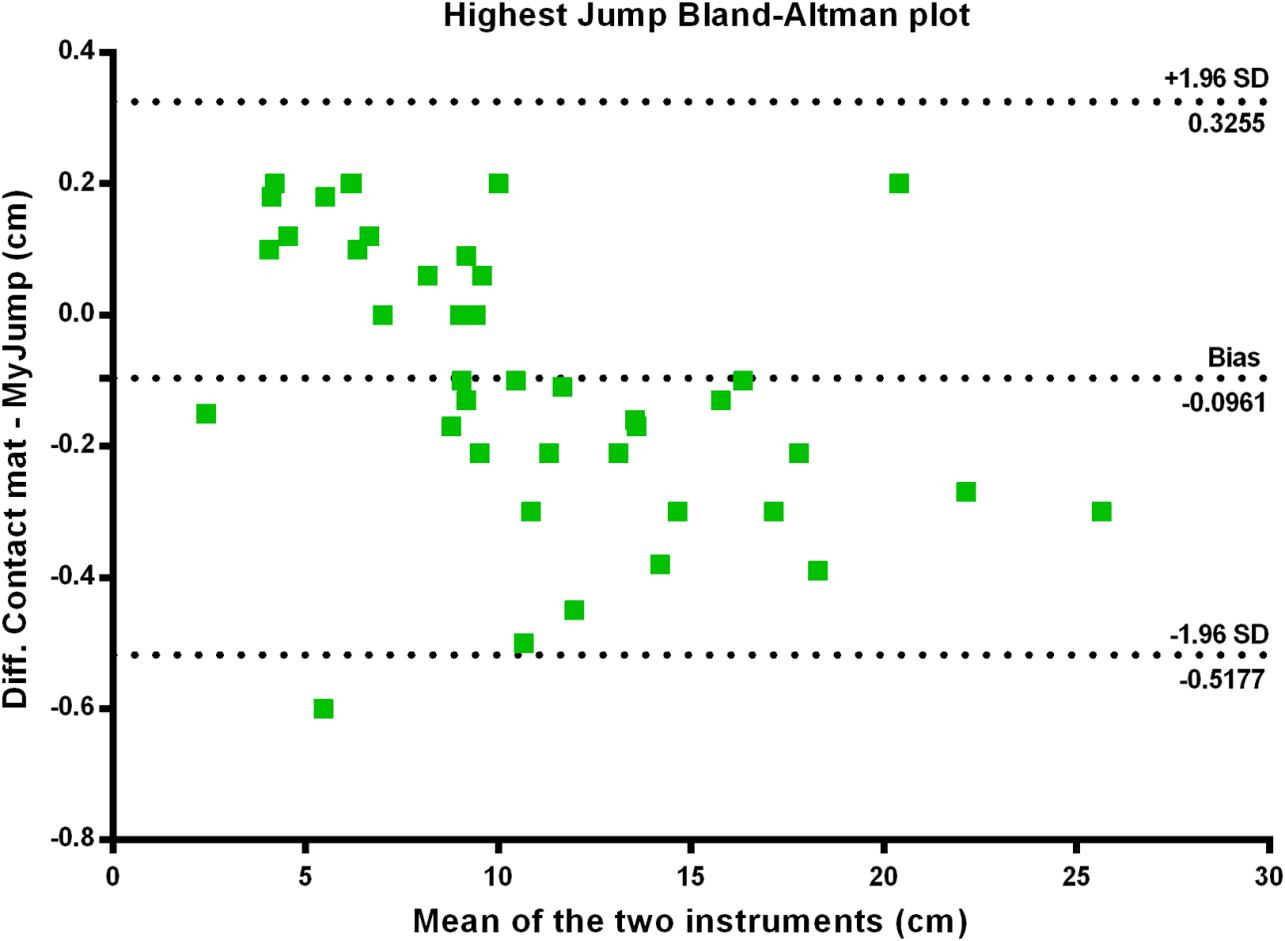
Highest jump Bland–Altman plot.

**Figure 5:**
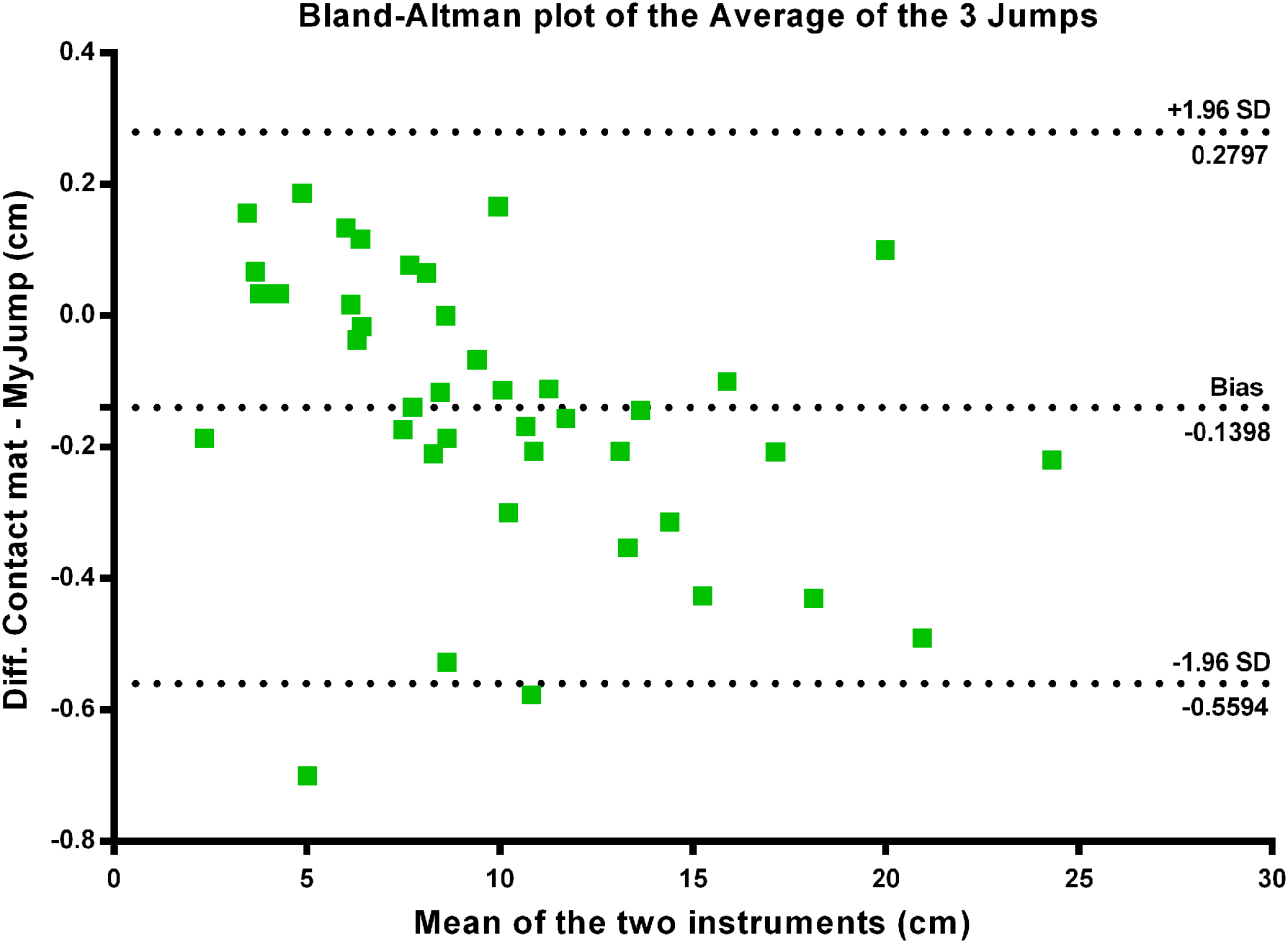
Bland–Altman plot of the average of three jumps.

**Figure 6:**
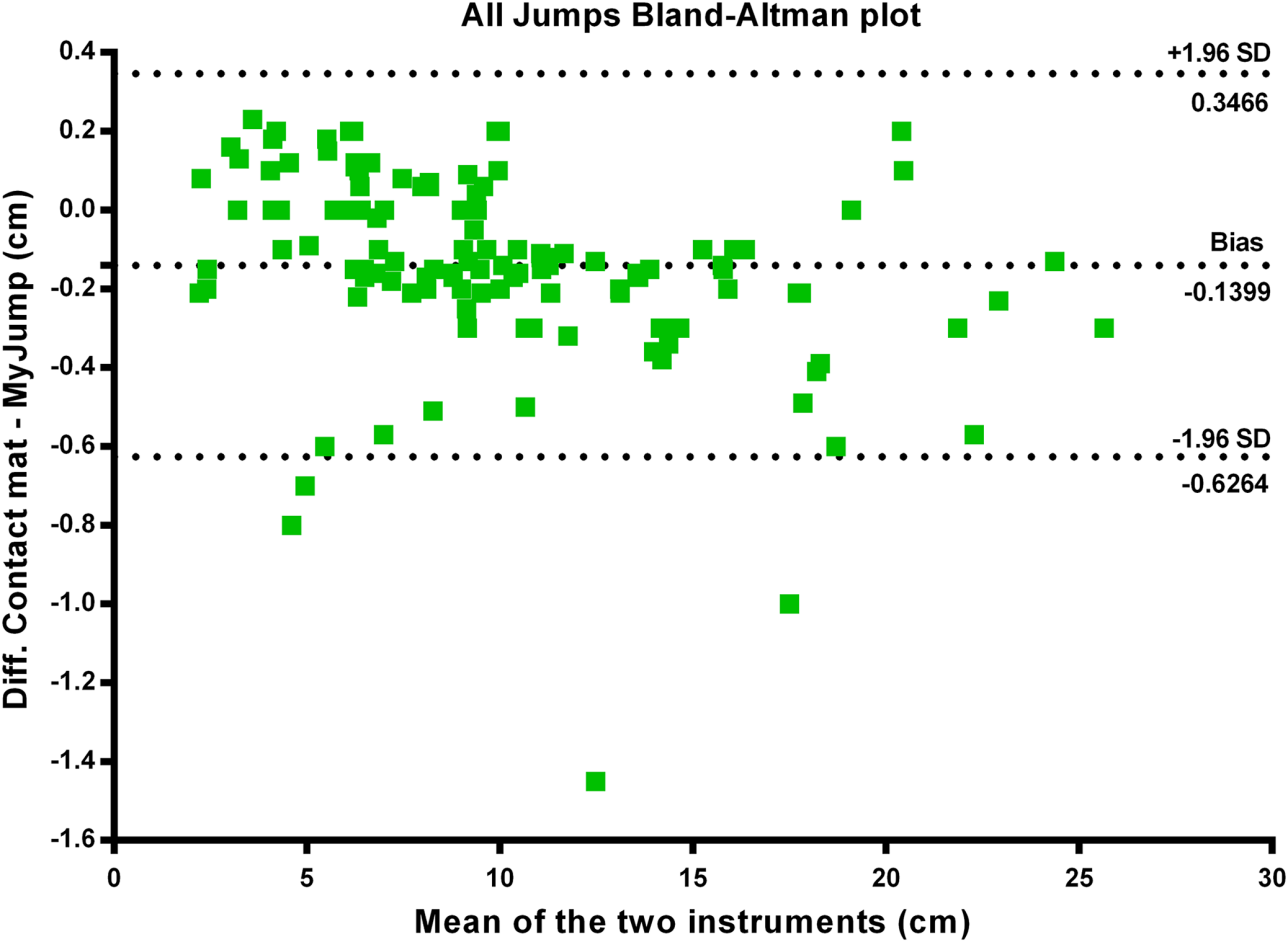
All jumps Bland–Altman plot.

There were nearly perfect correlations between the *My Jump App* and the contact mat heights using the highest jump (*r* = 0.999; *P* = 0.000) (see Fig. 1), the average of the three jumps (*r* = 0.999; *P* = 0.000) (see Fig. 2), as using all jumps (*r* = 0.999; *P* = 0.000) (see Fig. 3). The difference between the two methods using the highest jump was 0.096 cm with limits of agreement from 0.3255 cm to −0.5177 cm (see Fig. 4), while the difference between methods and limits of agreement of the methods using the average of the three jumps or all 123 jumps were higher (see Figs. 5 and 6, respectively). Good agreement was observed in all cases. However, the *t*-test showed significant differences between methods (see Table 1).

The *My Jump App* also showed excellent reliability levels (ICC = 0.948, TEM = 1.15, CV% = 10.096). The reliability results of both methods are presented in Table 2. Although the method presented high reliability, the analysis of variance employed for the calculation of the ICC showed a significant (*P* = 0.000) difference between the means, with higher mean height values for the third jump.

**Table 2:** Relative (ICC) and absolute (TEM and CV%) reliability of the two methods.

	ICC (95%CI)	TEM (cm)	CV%
Contact mat	0.949 (0.916–0.971)	1.128	10.111
*My Jump App*	0.948 (0.913–0.970)	1.150	10.096

**Note:**

ICC, intraclass correlation coefficient; TEM, typical error of measurement; CV%, TEM as a coefficient of variation.

## Discussion

The present investigation aimed to analyze the validity and reliability of the *My Jump App* for measuring jump height in the elderly. Our main findings showed a very good agreement of jump height recorded with the *My Jump App* with the reference method, as well as an almost perfect correlation. Further, intraday reliability levels were also very good.

To our knowledge, the *My Jump App* has never been used before for evaluating the vertical jump height in the elderly. Studies with other populations have demonstrated its validity and applicability (Balsalobre-Fernández, Glaister & Lockey, 2015; Carlos-Vivas et al., 2016; Driller et al., 2017). Previously, Balsalobre-Fernández, Glaister & Lockey (2015) validated this *App* for other populations and higher jumping values with a 120 Hz high-speed camera. The results of the current study extend its use to populations with lower values for jumping, which highlights the suitability of *My Jump App* for an elderly population. It is important to consider that in our study we used an iPhone model with a 240 Hz high-speed camera. The relatively higher video frequency (for a mobile/cell phone) could have contributed to the reduction in the measurement error of the *My Jump App*, especially for this population with such low jump height levels. Thus, practitioners in training and clinical settings may benefit from this easy-to-use, portable and affordable tool for this special population. Further studies should verify the minimum sampling rate of the mobile camera needed to maintain the results obtained in the current study, as well as the concurrent validity when considering other methods as force plates.

Our data presented in Bland–Altman plots (see Figs. 4–6) showed that the majority of the CMJ values are close to the mean of the differences between the two devices, thereby representing a high level of agreement (Bland & Altman, 1986). However, the slightly better agreement between methods for the highest jump (0.3255 to −0.5177 cm, 0.2797 to −0.5594 cm, and 0.3466 to −0.6264 cm, for highest jump height, average jump height, and all jumps height, respectively) would suggest that the highest jump height may be the best measure to identify performance changes in this population, adding further evidence to the previous suggestions by Claudino et al. (2017). However, there is an inability to calculate reliability values when using the highest jump. In addition, the almost near perfect correlation between methods confirms its concurrent validity (see Figs. 1–3). Meanwhile, the significant differences between methods indicates a systematic error with higher jumping heights with the *My Jump App* when compared to the contact mat (see Table 1). Therefore, comparisons between values from different studies obtained with different methods should consider this small bias. In addition, the *My Jump App* also showed excellent intraday absolute and relative reliability levels (ICC = 0.948, TEM = 1.15, CV% = 10.096) (see Table 2). However, the analysis of variance of the ICC revealed a significant difference for the last jump, with systematically higher jumping heights. From our observations, it could be speculated that the participants experienced greater confidence during the last attempts, therefore performing higher jumps. Another possibility is that an extensive dynamic warm-up was not provided prior to the jumps and thus the initial CMJ acted as a warm-up to potentiate the final CMJ. Further studies should elaborate on this issue, which could be also suggesting a possible learning or warm-up effect despite a previous familiarization session.

The vertical jump has been successfully used to evaluate individuals from a wide range of ages, showing lower values as age increases (Izquierdo et al., 1999; Runge et al., 2004). Our results are in agreement with the literature, showing lower mean values of the elderly in CMJ height when compared to younger populations (Izquierdo et al., 1999), and very similar to those obtained by others (Ditroilo et al., 2010; Farias et al., 2013) with a similar age group. An explanation for lower jump heights may be due to the decrease of muscle power during the ageing process as a consequence of the combination of a number of factors (e.g., neuromuscular, hormonal, genetic), which has been demonstrated to be more accentuated than the decrease in muscle mass (Alcazar et al., 2017) and maximal strength (Runge et al., 2004). CMJ height (cm) is a very simple measure directly linked to muscle power (Boullosa et al., 2018), which is one of the most important physical capacities related to the functional capacity (Pijnappels et al., 2008), risk of falls (Lord, Menz & Tiedemann, 2003), and independence of the elderly (Alcazar et al., 2017).

The main limitation of the methodology used by the *My Jump App* to measure jump height is the fact that the user has to manually select the frames in which the individual exactly performs the take-off and landing moments, thus making the measurement process subjective and time demanding in some cases. Moreover, another limitation could be the jumping technique of some participants who may be afraid of falling, therefore possibly compromising maximal performances in some cases. We could suggest that these issues could be easily overcome after familiarization of both practitioners and elderly. It is therefore unknown if more jump attempts would result in higher best jumps. On the other hand, there is an inability to calculate reliability values when using the highest jump, due to the need for at least two measurements for the calculations. Therefore, it was not possible to compare the TEM between the highest jump and the others used in the present study. Further studies should elaborate on this issue with more familiarization sessions to avoid a learning effect, which could not be excluded in the present investigation despite a previous familiarization session before evaluations.

The results of this study suggest that *My Jump* is a valid and reliable tool for measuring jump height in elderly people. Given its simplicity and practicality, it could be used by practitioners at different times and conditions for a rapid evaluation of the vertical jump capacity in the elderly. This would allow the evaluation of changes in functional capacity with a robust and simple test as CMJ. Furthermore, absolute and relative reliability measures demonstrated excellent results. Therefore, in addition to its low-cost compared with several reference methods available, the *My Jump App* can be considered a valid and reliable tool for calculating jump height in elderly people.

## Conclusion

The present study shows that the *My Jump App* presents a very high agreement with contact mat as reference method, demonstrating an excellent reliability. Therefore, in addition to its low-cost and simplicity, this method could be considered valid and reliable to calculate jump height in elderly people as in other populations. The highest jump and the average of jumps could be indistinctly used for identification of acute and chronic adaptations in vertical jumping capacity of the elderly.

## Supplemental Information

10.7717/peerj.5804/supp-1 Supplemental Information 1 Raw data.

## Additional Information and Declarations

### Data Availability

The following information was supplied regarding data availability: The raw data are provided in a Supplemental File.
